# Automated Evans index measurement using deep learning in acute subarachnoid hemorrhage: reliability, agreement with experts, and association with external ventricular drainage

**DOI:** 10.3389/fneur.2026.1786460

**Published:** 2026-06-19

**Authors:** Yanrui Cai, Huansong Wang, Huanhuan Yu, Yuxia Li, Baobao Meng, Qi Liu, Yuting Wang, Feiyu Qiao

**Affiliations:** 1 Department of Neurosurgery, Weifang People’s Hospital, Shandong Second Medical University, Weifang, China; 2 Department of Neurosurgery, Laixi People’s Hospital, Qingdao, China; 3 Department of Neurological Rehabilitation, Weifang Hospital of Traditional Chinese Medicine, Shandong Second Medical University, Weifang, China

**Keywords:** acute hydrocephalus, computed tomography, deep learning, Evans index, external ventricular drainage, subarachnoid hemorrhage

## Abstract

**Background:**

The Evans index (EI) is widely used to assess ventricular enlargement and support external ventricular drainage (EVD) decision-making after subarachnoid hemorrhage (SAH), but manual measurement is time-consuming and subject to inter-reader variability. The reliability of automated EI measurement in acute SAH remains insufficiently validated.

**Methods:**

In this retrospective cohort study, admission non-contrast CT scans from 364 patients with spontaneous SAH were analyzed using TotalSegmentator (TS), an open-source nnU-Net–based deep learning pipeline, to generate automated EI measurements. Each scan underwent two independent inference runs, and two neurosurgical experts independently performed manual measurements. Agreement between TS and expert measurements was evaluated for continuous EI values and EI > 0.30 classification. External ventricular drainage (EVD) placement was used as a pragmatic endpoint reflecting contemporaneous clinical decision-making. Prespecified subgroup analyses excluded frontal horn hematoma or frontal horn periventricular edema. Multivariable logistic regression assessed the association between EI and EVD placement after adjustment for key clinical covariates.

**Results:**

TS demonstrated excellent reproducibility between repeated inference runs (ICC = 0.996, 95% CI 0.996–0.997). Agreement between expert readers was high (ICC = 0.983, 95% CI 0.978–0.988). Between-method agreement between TS and expert EI measurements was good in the overall cohort (ICC = 0.76, 95% CI 0.73–0.81) and improved after exclusion of frontal horn hematoma (ICC = 0.87, 95% CI 0.85–0.89). For EI > 0.30 classification, TS identified more positive cases than expert assessment (29% vs. 17%), with moderate Cohen’s kappa (0.57, 95% CI 0.54–0.60). TS-derived EI demonstrated discrimination for EVD placement (AUC = 0.75, 95% CI 0.73–0.79), approaching expert-derived EI (AUC = 0.80, 95% CI 0.78–0.83). After covariate adjustment, TS-derived EI remained independently associated with EVD placement (adjusted OR = 1.09, 95% CI 1.03–1.17; *p* = 0.009).

**Conclusion:**

Automated EI measurement using TS provides reproducible and clinically informative assessment of ventricular enlargement on CT in acute SAH. Although threshold-sensitive disagreement occurred near EI = 0.30, automated EI showed meaningful agreement with expert assessment and remained independently associated with contemporaneous EVD decision-making. Further SAH-specific refinement may improve robustness in hemorrhage-related ventricular distortion.

## 1 Introduction

Subarachnoid hemorrhage (SAH) is an acute, life-threatening neurological emergency, most caused by rupture of an intracranial aneurysm, and predominantly affects individuals during their most productive years, resulting in a substantial individual and societal burden (1, 2). Acute hydrocephalus is among the most frequent and clinically consequential early complications of SAH. It can precipitate sustained intracranial hypertension and progressive impairment of consciousness, thereby increasing the risks of early mortality and long-term disability (3). In routine practice, timely recognition of acute hydrocephalus and prompt identification of patients who may require external ventricular drainage (EVD) are central to acute management and individualized intervention (4).

The Evans index (EI) is a classic linear marker of ventricular enlargement and remains widely used across neuroimaging settings, including normal-pressure hydrocephalus, pediatric hydrocephalus, and SAH-related hydrocephalus (5). EI is defined as the ratio of the maximum frontal horn width to the maximum inner skull diameter measured on the same axial plane at the level of the foramen of Monro (5, 6). Despite its conceptual simplicity, EI measurement is highly operator-dependent, relying on subjective plane selection and identification of anatomical landmarks. As a result, EI is susceptible to variability related to reader experience, image quality, and head positioning, with limited intra- and inter-observer reproducibility (7, 8). Even minor deviations in slice selection can lead to meaningful discrepancies, a limitation that becomes particularly salient in emergency workflows and across centers (6, 7, 9). These constraints motivate the need for objective, scalable approaches that can standardize EI measurement in neurocritical care settings.

In parallel, deep learning–based segmentation has advanced rapidly in medical image analysis (10–12) and has shown improved accuracy and robustness over traditional rule-based or geometric approaches for delineating intracranial structures, including the ventricular system and cerebrospinal fluid spaces (13–15). Self-configuring frameworks such as nnU-Net can achieve strong segmentation performance without extensive manual hyperparameter tuning and have been applied to a wide range of neuroimaging quantification tasks (16, 17). TotalSegmentator (TS), an open-source tool built on nnU-Net and pretrained on large-scale, multicenter computed tomography data, enables automated segmentation of multiple anatomical structures in a single inference and has the potential to support rapid, standardized quantification in time-sensitive clinical settings (18).

However, validating automated EI estimation in acute-phase SAH remains nontrivial. In contrast to chronic conditions with relatively preserved anatomy, acute SAH frequently involves subarachnoid blood, intraventricular hemorrhage, and intracerebral hematoma, often accompanied by brain edema, tissue shift, ventricular deformation, and blurred ventricular boundaries (15). These disease-specific features can impair segmentation fidelity and challenge the reliability of linear metrics derived from ventricular anatomy. Existing deep learning studies of ventricular or cerebrospinal fluid segmentation have largely focused on chronic hydrocephalus, particularly normal pressure hydrocephalus (NPH), or pediatric populations without hemorrhage, where hemorrhage burden is minimal and anatomical distortion is relatively limited, often with small sample sizes and more regular anatomical structures (19–21). Moreover, work in SAH cohorts has predominantly emphasized volumetric indices. These studies have less frequently examined agreement for clinically established linear metrics, such as the Evans index, or linked automated measurements to real-world clinical actions, such as EVD placement. Consequently, high-quality evidence remains limited regarding the reproducibility and agreement with expert assessment of automated Evans index measurement. In addition, its practical clinical relevance using open-source deep learning models in the complex imaging context of acute SAH has not been well established.

Against this background, we conducted a single-center retrospective cohort study to evaluate the performance of TS for automated EI measurement on admission non-contrast CT scans of patients with SAH. Specifically, we aimed to: (1) quantify the reproducibility of automated EI measurements and compare them with repeated expert measurements, for both continuous EI values and threshold-based classification (EI > 0.30); (2) assess the discriminative utility of automated EI with EVD as a pragmatic clinical endpoint, and directly compare performance with expert assessment; and (3) perform prespecified subgroup analysis to evaluate key disease-related sources of measurement interference and its impact on agreement and clinical discrimination. By providing systematic validation in a hemorrhagic and anatomically complex setting, this study seeks to inform the potential role of open-source deep learning tools as rapid, standardized adjuncts for hydrocephalus assessment in emergency neuroimaging workflows.

## 2 Materials and methods

This study was conducted and reported in accordance with the Guidelines for Reporting Reliability and Agreement Studies (GRRAS) and the Checklist for Artificial Intelligence in Medical Imaging (CLAIM) (22, 23).

### 2.1 Study design and population

This single-center retrospective observational study consecutively enrolled adult patients admitted to the Department of Neurosurgery at Weifang People’s Hospital between January 2020 and October 2025 with spontaneous SAH confirmed on non-contrast head CT. All imaging and clinical data were locked on October 31, 2025, and anonymized thereafter. This retrospective study was conducted in accordance with the Declaration of Helsinki and approved by the Institutional Review Board of Weifang People’s Hospital (Approval No. KYLL20250507-3), which waived the requirement for informed consent owing to the retrospective design and use of anonymized data.

The first non-contrast head CT obtained at presentation was defined as the index CT and served as the basis for EI measurement and all subsequent analyses. To ensure that EI measurements reflected ventricular status at the time of clinical decision-making regarding EVD, rather than serving as a delayed predictor of subsequent ventricular deterioration, patients who underwent EVD were included only if EVD was performed within 3 h after the index CT; timing was verified using operative records. A relatively strict time window was selected to minimize temporal bias caused by evolving hydrocephalus, clot redistribution, or intracranial pressure changes that could alter ventricular morphology between imaging and intervention. For patients who did not undergo EVD, the index CT was the admission scan for all cases, and non-EVD status was confirmed throughout hospitalization, ensuring that ventricular status was not confounded by delayed intervention.

Inclusion criteria were: (1) spontaneous SAH confirmed on non-contrast CT; (2) age ≥18 years; (3) availability of an admission index CT with a slice thickness ≤5 mm; and (4) complete clinical data with definitive documentation of EVD status. Exclusion criteria were: Exclusion criteria were: (1) severe CT artifacts precluding reliable delineation of the ventricular system; (2) marked ventricular distortion due to prior major cranial surgery or substantial parenchymal pathology; (3) traumatic SAH; (4) hydrocephalus secondary to non-SAH etiologies; (5) EVD performed more than 3 h after the index CT; and (6) missing key imaging or clinical information.

An *a priori* sample size calculation was performed using the presize package in R. Assuming an intraclass correlation coefficient (ICC) of 0.75–0.90 and targeting a 95% confidence interval width of ≤0.10, the estimated sample size requirement ranged from approximately 60 to 300 patients. The final cohort comprised 364 patients, exceeding the prespecified minimum needed to achieve the desired level of precision.

### 2.2 Clinical and imaging data collection

Demographic characteristics, admission GCS scores, Hunt–Hess grades, aneurysm status, and major complications were extracted from electronic medical records and independently verified by two neurosurgeons. Decisions regarding EVD placement were made independently by the attending or senior neurosurgeon based on routine clinical judgment and were not informed by any automated or study-related manual EI measurements. Indications for EVD generally included radiological evidence of acute hydrocephalus or progressive ventricular enlargement, neurological deterioration, suspected intracranial hypertension, and overall clinical assessment. EVD placement was used as a pragmatic real-world clinical endpoint reflecting acute hydrocephalus requiring surgical intervention.

### 2.3 Evans index measurement

#### 2.3.1 Automated measurement

Automated EI measurement was performed using TotalSegmentator (version 2.11.0), an open-source deep learning pipeline built on the nnU-Net framework. Original CT images were converted from DICOM to NIfTI format with Mango (version 4.1) prior to processing. Model inference was performed using an NVIDIA GeForce RTX 3060 GPU with 12 GB VRAM, with all parameters set to default values. TS applies internal preprocessing steps—including intensity normalization, resampling to a standardized voxel spacing, and automated skull stripping—without requiring user-defined resampling or parameter tuning.

Images were rigidly registered using the built-in registration pipeline implemented within TS, with the integrated CT brain atlas serving as the reference template and the orbitomeatal line used as the spatial reference plane (24). The model automatically segmented the bilateral frontal horns of the lateral ventricles and applied post-processing steps, including connected-component filtering, removal of small isolated components, and brain-mask–based structural correction, to improve segmentation robustness. The EI was calculated as the ratio of frontal horn width to inner calvarial diameter, with both measurements obtained from the same atlas-registered axial slice and without independent slice optimization.

**Figure 1:**
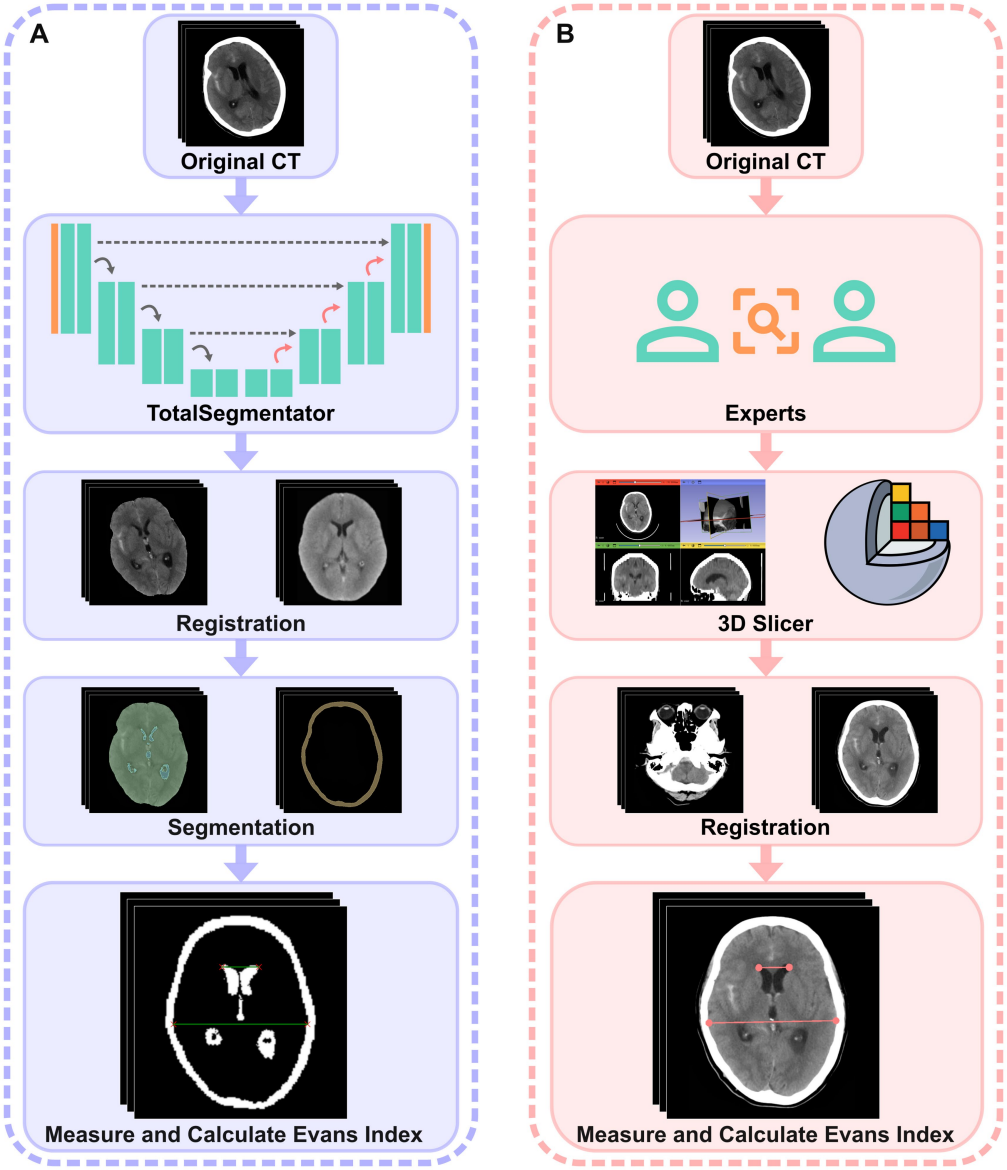
Automated and expert-based workflows for Evans index (EI) measurement. **(A)** Automated workflow. Admission non-contrast head CT images are processed using an open-source deep learning pipeline (TotalSegmentator) to automatically calculate the Evans index. The pipeline is executed twice for each scan to assess reproducibility. **(B)** Expert-based workflow. Two neurosurgical experts independently perform Evans index measurements using 3D Slicer after standardized image reformatting.

**Figure 2:**
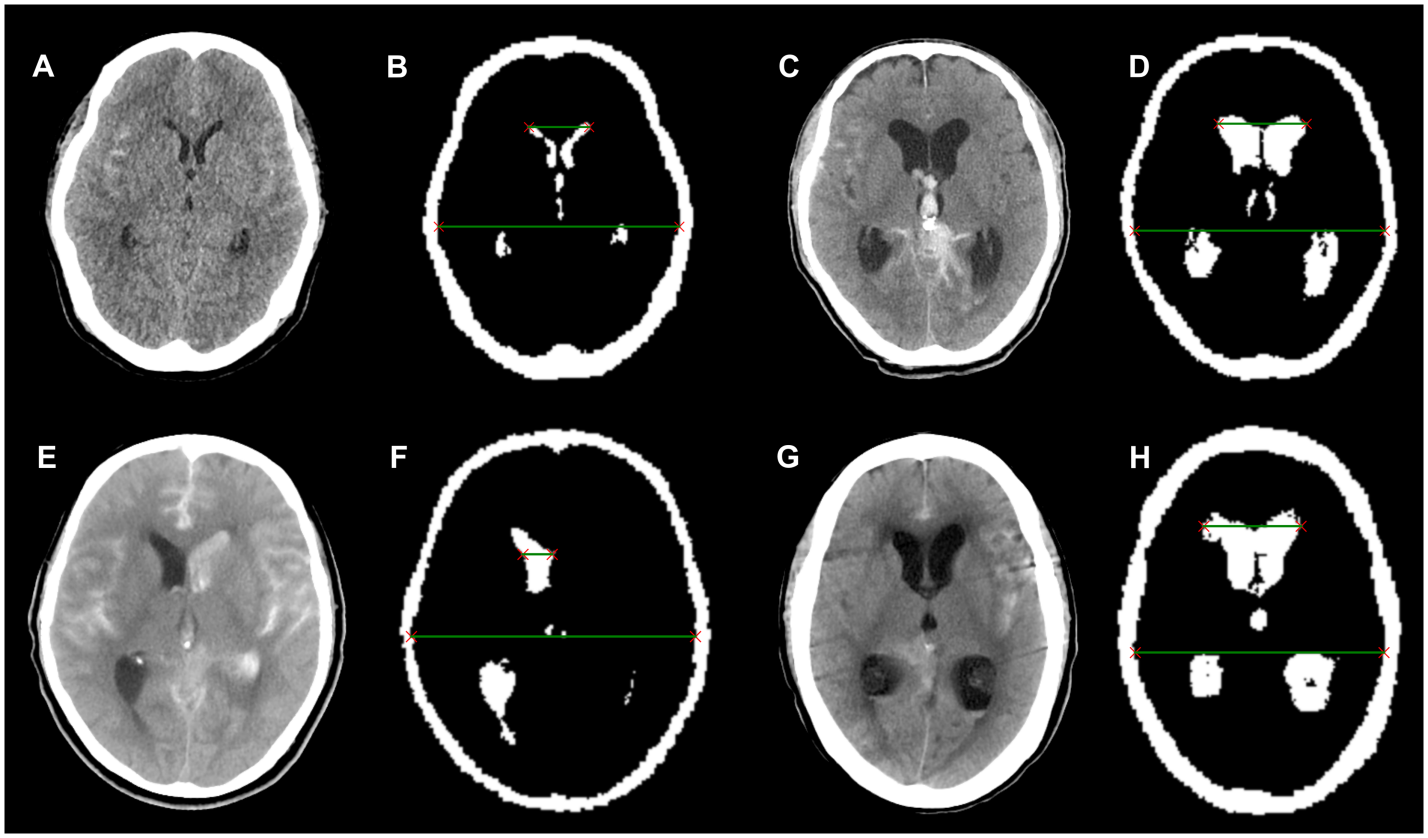
Representative examples of automated Evans index (EI) measurement under different ventricular and hemorrhagic conditions. Axial non-contrast head CT images **(A,C,E,G)** and the corresponding automated segmentations with Evans index measurement overlays generated by TotalSegmentator **(B,D,F,H)** are shown. **(A,B)** A case without ventricular enlargement. **(C,D)** A case with ventricular enlargement without frontal horn hemorrhage. **(E,F)** A case with left frontal horn casting by intraventricular hemorrhage. **(G,H)** A case with ventricular enlargement accompanied by periventricular exudation.

To assess pipeline stability, each scan was processed twice to generate repeated automated EI measurements. No test-time augmentation was used, and no model fine-tuning or retraining was performed, consistent with an off-the-shelf deployment setting. Because inference was deterministic under fixed model parameters and preprocessing conditions, repeated runs were expected to produce identical or near-identical results; repeated processing was performed primarily to confirm pipeline stability and reproducibility. The automated workflow is illustrated in Figure 1A, with representative examples shown in Figure 2.

#### 2.3.2 Manual measurement

Manual EI measurements were performed using 3D Slicer (version 5.10.0) by two neurosurgeons with extensive clinical experience. Images were manually registered in 3D Slicer using the same CT brain atlas template applied in the TS-based workflow to ensure consistency between automated and expert measurements. Images were reviewed using a standardized brain window (window width 80 Hounsfield units; window level 40 Hounsfield units). Multiplanar reconstruction was used to align image planes parallel to the orbitomeatal line. On the axial slice passing through the foramen of Monro, the maximum widths of both frontal horns and the maximum inner table diameter of the calvarium were measured, and EI was calculated accordingly. The manual measurement workflow is illustrated in Figure 1B.

### 2.4 Reproducibility and agreement analysis

To evaluate reproducibility within each measurement approach, automated EI measurements were generated from two independent inference runs, whereas expert reproducibility was assessed between two independent neurosurgical readers. For between-method agreement analyses, comparisons were performed using the mean EI values derived from the two TS runs and the two expert measurements, respectively.

Agreement for continuous EI values was assessed using Spearman correlation coefficients, intraclass correlation coefficients (ICC), Lin’s concordance correlation coefficients (Lin’s CCC), and Bland–Altman analysis. Threshold-based agreement for EI > 0.30 was evaluated using Cohen’s kappa, Gwet’s AC1, and prevalence-adjusted bias-adjusted kappa (PABAK). Multiple agreement coefficients were reported because Cohen’s kappa may be influenced by class imbalance and prevalence effects, particularly when positive classifications are relatively infrequent. Gwet’s AC1 and PABAK were therefore additionally included to provide more robust estimates of threshold-based agreement under imbalanced classification conditions (25). Sensitivity, specificity, F1 score, and Matthews correlation coefficient were additionally reported to characterize classification performance.

In addition, an exploratory sensitivity analysis was conducted focusing on the EI threshold of 0.30. Agreement metrics and classification performance were recalculated across a range of values surrounding this cutoff to assess the robustness of threshold-based classification. A density-based analysis was further performed to characterize the distribution of discordant cases around the 0.30 decision boundary.

### 2.5 Clinical discrimination analysis

To evaluate the clinical relevance of EI measurements, discriminative performance for EVD placement was assessed using receiver operating characteristic analysis, with area under the curve reported as the primary metric. Patients who underwent EVD placement during clinical management were considered positive cases. Sensitivity, specificity, accuracy, and F1 score were also calculated to characterize performance across clinically relevant operating points.

Because EVD placement decisions are influenced by multiple clinical and radiographic factors, additional multivariable logistic regression analyses were performed to assess whether the association between EI measurements and clinically performed EVD placement remained significant after adjustment for potential confounders. Separate models were constructed using TS and expert measurements, adjusting for age, sex, admission GCS score, Hunt–Hess grade, and modified Fisher grade. ORs with 95% CIs and *p* value were reported.

### 2.6 Subgroup analysis

Because accurate EI measurement depends on clear delineation of frontal horn boundaries, two representative imaging-related interfering factors were prespecified: (1) frontal horn hematoma, which may obscure ventricular margins and impair segmentation fidelity, and (2) frontal horn periventricular edema, which may lead to inadvertent inclusion of extra-ventricular low-density regions during automated segmentation.

After excluding patients with frontal horn hematomas and, separately, patients with frontal horn periventricular edema, agreement analyses between TS and experts EI measurements were repeated to quantify the impact of these disease-specific confounders on continuous agreement and threshold-based classification performance. Clinical discrimination analyses for EVD placement were repeated only for the frontal horn hematoma subgroup because frontal horn periventricular edema were frequently associated with hydrocephalus and EVD placement. Excluding these patients would substantially alter the clinical composition of the cohort and reduce the interpretability of EVD-related analyses.

### 2.7 Discrepancy analysis between TS and expert EI measurements

To further investigate potential causes of disagreement between automated and expert EI measurements, cases with an absolute difference greater than 0.05 between TS and expert measurements were retrospectively reviewed. Imaging findings potentially contributing to measurement discrepancies were independently assessed by two neurosurgical experts, and the factor considered most directly related to the discrepancy in each case was determined by consensus. Discrepancy patterns were categorized into predefined imaging features, including frontal horn hemorrhage, hemorrhage in other ventricular regions, frontal horn periventricular edema, intracerebral hemorrhage, and interhemispheric fissure hematoma.

### 2.8 Statistical analysis

Statistical analyses were performed using R (version 4.4.2). Continuous variables are presented as mean ± standard deviation or median (interquartile range), and categorical variables as counts and percentages. Between-group comparisons used independent-samples t-tests for normally distributed continuous variables and Mann–Whitney U tests for non-normally distributed variables. Categorical variables were compared using chi-square or Fisher’s exact tests, as appropriate. All statistical tests were two-sided, with a significance threshold of 0.05.

## 3 Results

### 3.1 Study population

**Figure 3:**
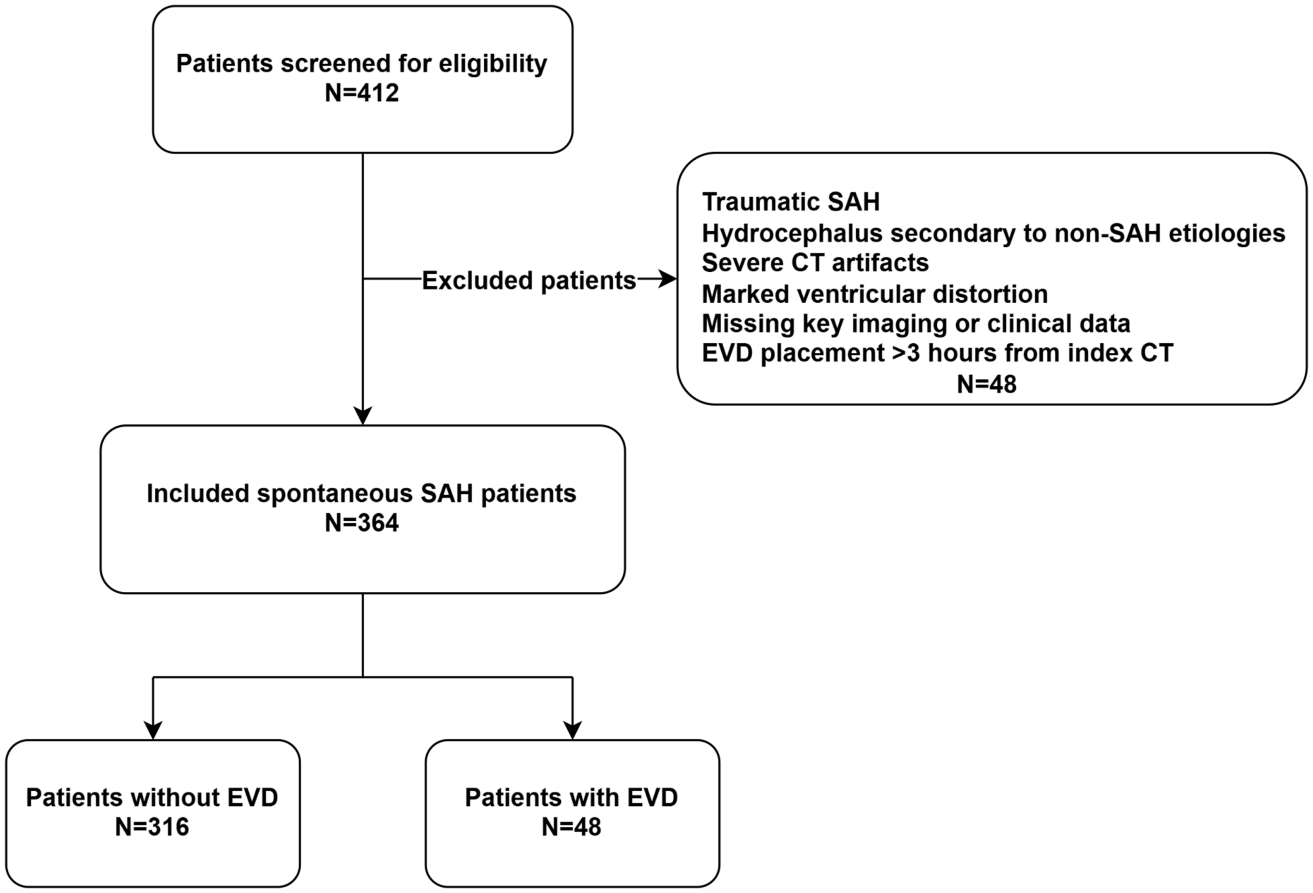
Flowchart of patient selection and subgroup analysis. Flowchart illustrating patient screening, exclusion criteria, final cohort inclusion, EVD stratification.

A total of 364 patients with spontaneous subarachnoid hemorrhage were included (Figure 3). The median age was 58 years (interquartile range [IQR], 51–67), and 146 patients (40%) were men. The median admission GCS score was 13 (IQR, 11–15), and Hunt–Hess grades II (34%) and III (35%) were most common. On imaging, 260 patients (71%) had an intracranial aneurysm, 17 (4.7%) had frontal horn intraventricular hemorrhage, and 28 (7.7%) had concomitant intracerebral hematoma (Table 1).

**Table 1:** Baseline characteristics of patients with subarachnoid hemorrhage according to external ventricular drainage status.

Variable	Overall (*N* = 364)	Non-EVD (*N* = 316)	EVD (*N* = 48)	*p* value
Demographics				
Age (years)	58 (51, 67)	58 (50, 65)	63 (56, 69)	0.008
Gender, *n* (%)	146 (40%)	129 (41%)	17 (35%)	0.5
Clinical characteristics				
Intracranial aneurysm, *n* (%)	260 (71%)	220 (70%)	40 (83%)	0.050
GCS	13 (11, 15)	13 (12, 15)	10 (6, 13)	<0.001
Hunt-Hess				<0.001
1	40 (11%)	38 (12%)	2 (4.2%)	
2	125 (34%)	119 (38%)	6 (13%)	
3	127 (35%)	112 (35%)	15 (31%)	
4	61 (17%)	43 (14%)	18 (38%)	
5	11 (3.0%)	4 (1.3%)	7 (15%)	
Radiological characteristics				
Frontal horn hemorrhage, *n* (%)	17 (4.7%)	9 (2.8%)	8 (17%)	<0.001
Intracerebral hemorrhage, *n* (%)	28 (7.7%)	20 (6.3%)	8 (17%)	0.020

Among the 364 patients, 48 underwent EVD. Compared with the non-EVD group, patients receiving EVD were older (median 63 years [IQR, 56–69] vs. 58 years [IQR, 50–65]), had lower admission GCS scores (median 10 [IQR, 6–13] vs. 13 [IQR, 12–15]), and more frequently presented with Hunt–Hess grades IV–V (53% vs. lower in non-EVD). The EVD group also had a higher prevalence of intracranial aneurysm (83% vs. 70%), frontal horn intraventricular hemorrhage (17% vs. 2.8%), and intracerebral hematoma (17% vs. 6.3%). Sex distribution did not differ significantly between groups (Table 1).

### 3.2 Within-method reproducibility

Within-method reproducibility results are summarized in Table 2. For the automated approach, correlation between two independent TS inference runs was high (Spearman *ρ* = 0.995, 95% CI 0.994–0.995), with excellent agreement (ICC = 0.996, 95% CI 0.996–0.997; Lin’s CCC = 0.996, 95% CI 0.995–0.997). For the expert approach, agreement between the two independent neurosurgical readers was similarly high (Spearman ρ = 0.982, 95% CI 0.980–0.984; ICC = 0.983, 95% CI 0.978–0.988; Lin’s CCC = 0.984, 95% CI 0.976–0.988).

**Table 2:** Within-method reproducibility of automated and expert Evans index measurements.

Metric	Expert measurements	TS measurements
Continuous		
Spearman	0.982 (0.980, 0.984)	0.995 (0.994, 0.995)
ICC	0.983 (0.978, 0.988)	0.996 (0.996, 0.997)
Lin’s CCC	0.984 (0.976, 0.988)	0.996 (0.995, 0.997)
Categorical		
Cohen’s kappa	0.86 (0.84, 0.89)	0.95 (0.94, 0.96)
Gwet’s AC1	0.95 (0.94, 0.96)	0.96 (0.95, 0.97)
PABAK	0.92 (0.91, 0.94)	0.96 (0.94, 0.97)

**Figure 4:**
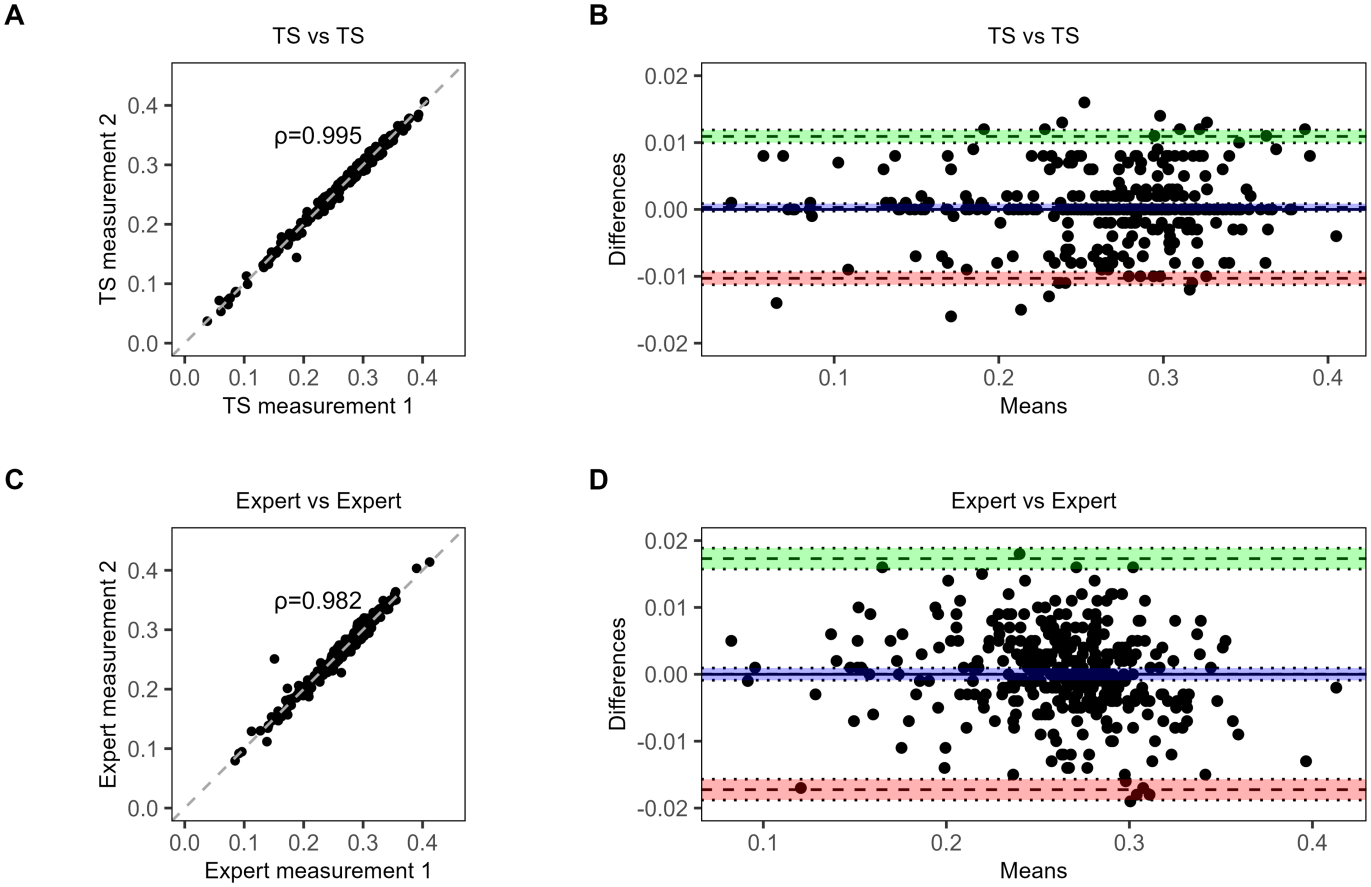
Within-method reproducibility of Evans index (EI) measurements for automated and expert assessments. Scatter plots **(A,C)** and Bland–Altman plots **(B,D)** illustrate the agreement between repeated Evans index measurements obtained using the same method. **(A,B)** Agreement between two independent automated measurements generated by TotalSegmentator. **(C,D)** Agreement between repeated measurements performed by neurosurgical experts.

For threshold-based classification (EI > 0.30), agreement between the two TS runs was excellent (Cohen’s kappa = 0.95, 95% CI 0.94–0.96; Gwet’s AC1 = 0.96, 95% CI 0.95–0.97; PABAK = 0.96, 95% CI 0.94–0.97). Agreement between the two expert readers was also high (Cohen’s kappa = 0.86, 95% CI 0.84–0.89; AC1 = 0.95, 95% CI 0.94–0.96; PABAK = 0.92, 95% CI 0.91–0.94). Bland–Altman plots demonstrated narrow between-measurement differences without systematic bias for either approach (Figure 4).

### 3.3 Between-method agreement (TS vs. experts)

**Figure 5:**
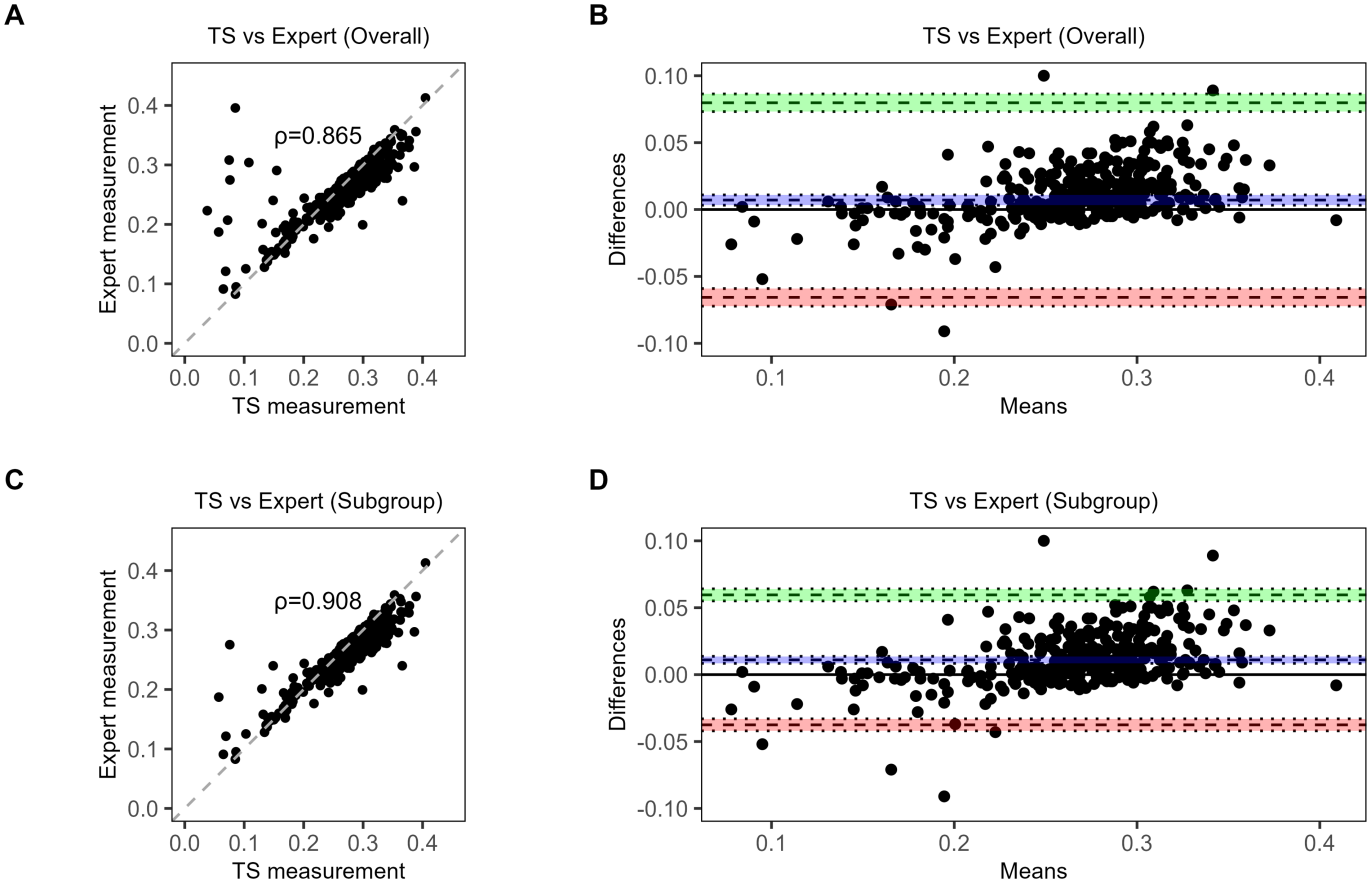
Between-method agreement of Evans index (EI) measurements between automated and expert assessments. Scatter plots **(A,C)** and Bland–Altman plots **(B,D)** illustrate the agreement between automated Evans index measurements generated by TotalSegmentator and expert-derived measurements. **(A,B)** Agreement in the overall cohort. **(C,D)** Agreement in the prespecified subgroup excluding cases with frontal horn hemorrhage.

Between-method agreement results are summarized in Table 3. In the overall cohort, automated and expert EI measurements were strongly correlated (Spearman *ρ* = 0.87, 95% CI 0.85–0.88), with good agreement (ICC = 0.76, 95% CI 0.73–0.81; Lin’s CCC = 0.77, 95% CI 0.73–0.79). Bland–Altman analysis demonstrated a small mean bias (−0.007) with 95% limits of agreement of approximately −0.08 to 0.07 (Figure 5).

**Table 3:** Between-method agreement between automated and expert Evans index measurements in the overall cohort and in the subgroup excluding frontal horn hematoma and excluding frontal horn periventricular edema.

Metric	TS vs expert (overall cohort)	TS vs expert (excluding frontal horn hematoma)	TS vs expert (excluding frontal horn periventricular edema)	*p*
Continuous				
Spearman	0.87 (0.85, 0.88)	0.91 (0.90, 0.92)	0.86(0.84,0.88)	<0.001
ICC	0.76 (0.73, 0.81)	0.87 (0.85, 0.89)	0.77(0.73,0.81)	<0.001
Lin’s CCC	0.77 (0.73, 0.79)	0.87 (0.85, 0.89)	0.76(0.73,0.82)	<0.001
Categorical				
Cohen’s kappa	0.57 (0.54, 0.60)	0.57 (0.55, 0.61)	0.62(0.58,0.67)	<0.001
Gwet’s AC1	0.74 (0.71, 0.77)	0.75 (0.73, 0.77)	0.83(0.81,0.85)	<0.001
PABAK	0.68 (0.65, 0.69)	0.69 (0.67, 0.72)	0.77(0.75,0.79)	<0.001

For threshold-based classification using EI > 0.30, positive cases were more frequently identified by TS than by expert assessment (29% vs. 17%). Agreement between TS and experts was moderate to good (Cohen’s kappa = 0.57, 95% CI 0.54–0.60; Gwet’s AC1 = 0.74, 95% CI 0.71–0.77; PABAK = 0.68, 95% CI 0.65–0.69).

**Figure 6:**
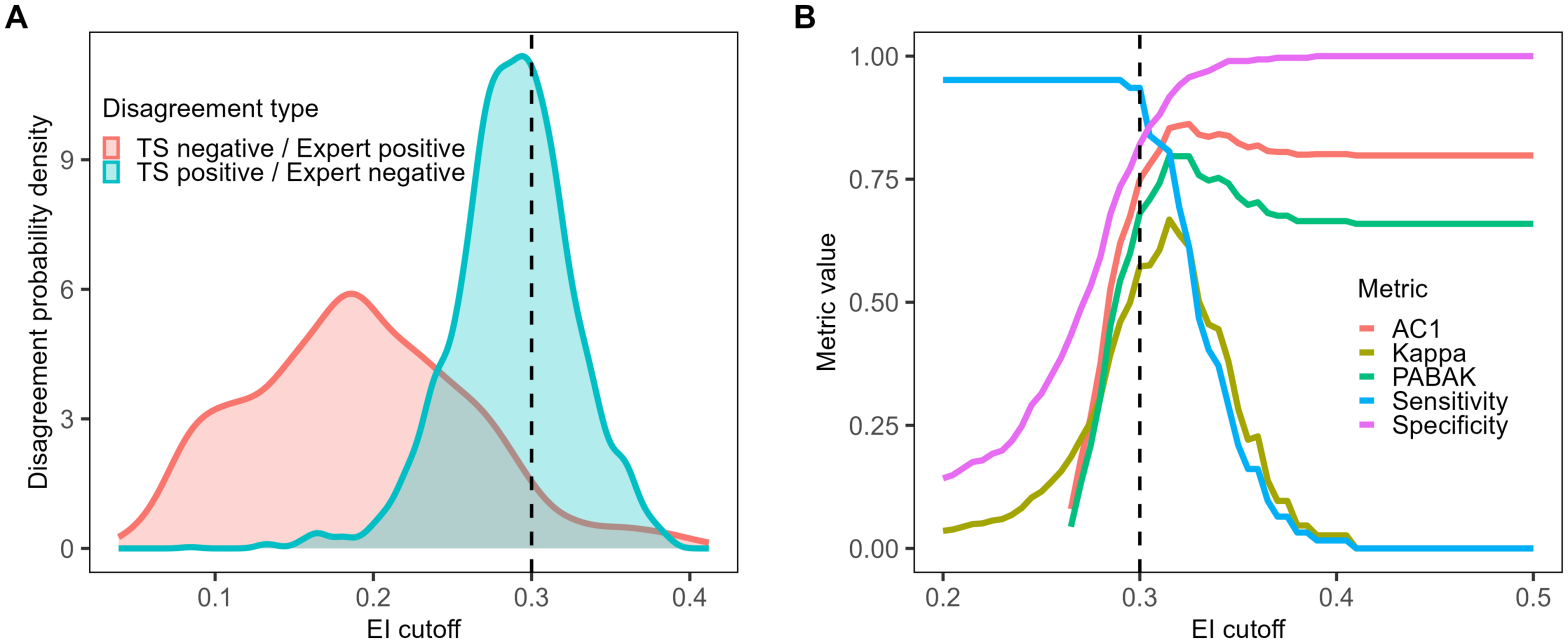
Threshold-sensitive disagreement and performance analysis across alternative Evans index (EI) cutoffs. Density plot **(A)** and threshold-performance curves **(B)** illustrate disagreement patterns and changes in agreement and diagnostic performance across alternative Evans index thresholds. **(A)** Density distribution of discordant binary classifications between TS-derived and expert-derived EI measurements near the conventional EI cutoff of 0.30. **(B)** Threshold-performance analysis showing changes in Cohen’s kappa, Gwet’s AC1, PABAK, sensitivity, specificity, and positive classification prevalence across alternative EI cutoffs.

Disagreement-density analysis showed that discordant classifications were concentrated around the EI = 0.30 cutoff (Figure 6A), indicating that small differences between automated and expert measurements were sufficient to alter binary classification. Directional disagreement was asymmetric around the cutoff: cases above the threshold were more often classified as positive by TS but negative by expert assessment, whereas cases below the threshold more often showed the opposite pattern.

Threshold sensitivity analysis further demonstrated that increasing the EI cutoff increased specificity while reducing sensitivity, whereas lowering the cutoff produced the opposite pattern (Figure 6B). Agreement metrics varied non-linearly across thresholds. Cohen’s kappa reached its maximum at approximately EI = 0.32 and gradually declined at higher thresholds. Gwet’s AC1 and PABAK showed similar overall trends but remained consistently higher than Cohen’s kappa across most cutoff values, reflecting greater robustness to prevalence imbalance.

### 3.4 Subgroup analysis

Exclusion of patients with frontal horn hematoma (excluded *n* = 17; remaining *n* = 347) improved agreement between TS and expert measurements for continuous EI values. Spearman correlation increased to 0.91 (95% CI 0.90–0.92), with an ICC of 0.87 (95% CI 0.85–0.89) and Lin’s CCC of 0.87 (95% CI 0.85–0.89). Bland–Altman analysis showed a mean bias of −0.011 with narrower limits of agreement (approximately −0.060 to 0.038) (Figure 4). For EI > 0.30 classification in this subgroup, agreement metrics remained similar to those in the overall cohort (Cohen’s kappa = 0.57, 95% CI 0.55–0.61; AC1 = 0.75, 95% CI 0.73–0.77; PABAK = 0.69, 95% CI 0.67–0.72) (Table 3).

By comparison, removal of patients with frontal horn periventricular edema (excluded *n* = 36; remaining *n* = 328) had little effect on continuous agreement between TS and expert measurements. Spearman correlation was 0.86 (95% CI 0.84–0.88), with an ICC of 0.77 (95% CI 0.73–0.81) and Lin’s CCC of 0.76 (95% CI 0.73–0.82) (Table 3). However, threshold-based agreement for EI > 0.30 classification improved in this subgroup. Cohen’s kappa increased to 0.62 (95% CI 0.58–0.67), Gwet’s AC1 to 0.83 (95% CI 0.81–0.85), and PABAK to 0.77 (95% CI 0.75–0.79).

### 3.5 Clinical discrimination for EVD placement

**Figure 7:**
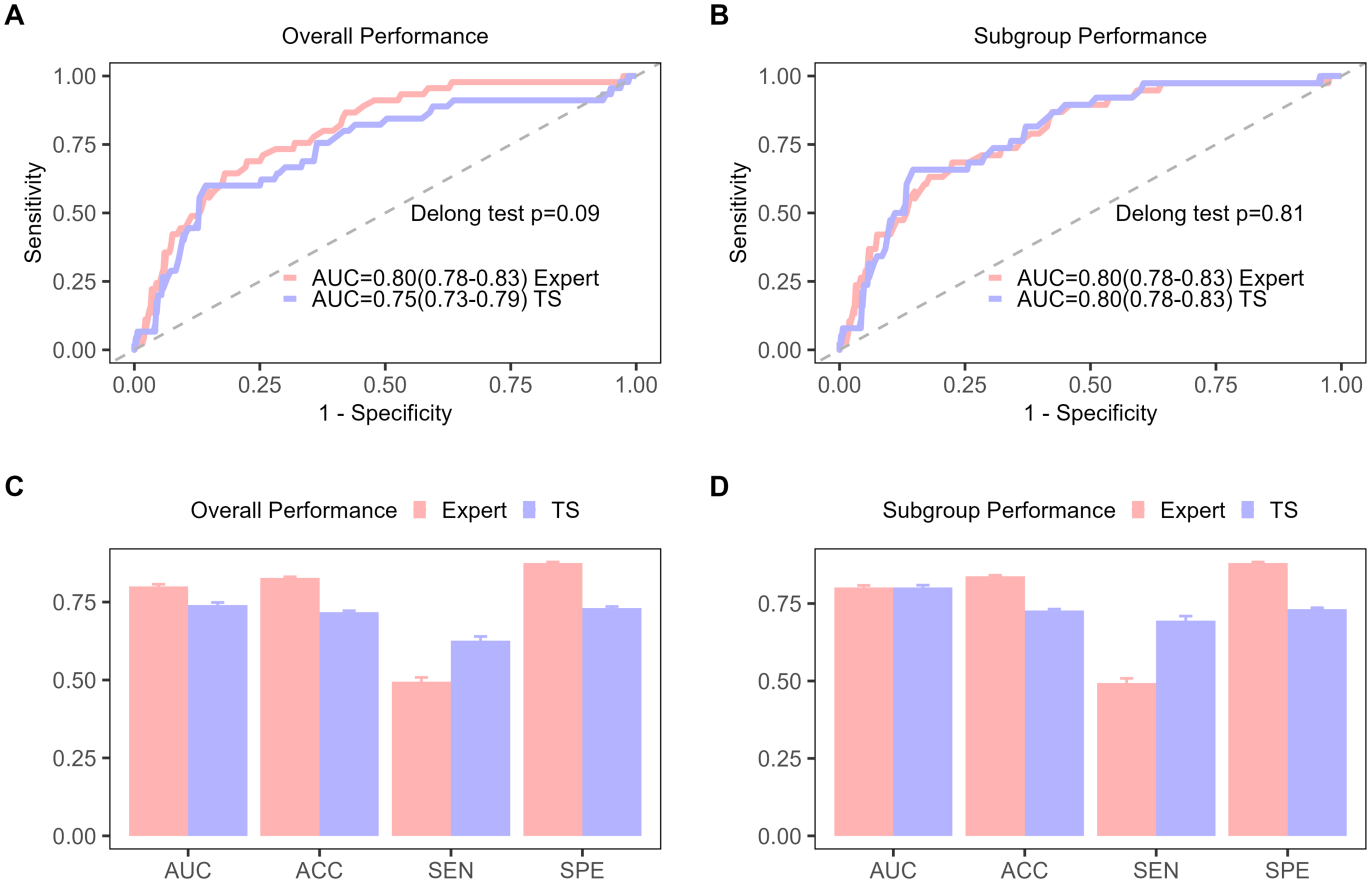
Discrimination performance of expert and automated Evans index measurements for EVD prediction. Receiver operating characteristic (ROC) curves and summary diagnostic metrics comparing expert-derived and automated Evans index (EI) measurements generated by TotalSegmentator (TS) for predicting external ventricular drainage (EVD) in patients with subarachnoid hemorrhage. **(A)** ROC curves in the overall cohort. **(B)** ROC curves in the prespecified subgroup excluding frontal horn hematoma. **(C)** AUC, accuracy (ACC), sensitivity (SEN), and specificity (SPE) in the overall cohort. **(D)** Corresponding performance metrics in the subgroup excluding frontal horn hematoma.

Clinical discrimination results are summarized in Figure 7 and Table 4. In the overall cohort, the area under the receiver operating characteristic curve for EVD placement was 0.75 (95% CI 0.73–0.79) for TS-derived EI and 0.80 (95% CI 0.78–0.83) for expert-derived EI. TS measurements yielded an F1 score of 0.36 (95% CI 0.33–0.38), sensitivity of 0.62 (95% CI 0.58–0.67), and specificity of 0.73 (95% CI 0.72–0.75), whereas expert measurements yielded an F1 score of 0.42 (95% CI 0.38–0.45), sensitivity of 0.49 (95% CI 0.44–0.54), and specificity of 0.88 (95% CI 0.86–0.89).

**Table 4:** Clinical discrimination performance of automated and expert Evans index measurements for external ventricular drainage placement.

Metric	Expert (overall cohort)	TS (overall cohort)	Expert (excluding frontal horn hematoma)	TS (excluding frontal horn hematoma)
F1-Score	0.42 (0.38, 0.45)	0.36 (0.33, 0.38)	0.40 (0.36, 0.44)	0.36 (0.33, 0.39)
Sensitivity	0.49 (0.44, 0.54)	0.62 (0.58, 0.67)	0.50 (0.45, 0.55)	0.70 (0.63, 0.74)
Specificity	0.88 (0.86, 0.89)	0.73 (0.72, 0.75)	0.88 (0.87, 0.89)	0.73 (0.71, 0.75)
Accuracy	0.83 (0.82, 0.84)	0.72 (0.70, 0.74)	0.84 (0.83, 0.85)	0.73 (0.71, 0.74)
PPV	0.36 (0.33, 0.39)	0.25 (0.23, 0.27)	0.34 (0.30, 0.38)	0.24 (0.22, 0.26)
NPV	0.92 (0.92, 0.93)	0.93 (0.92, 0.94)	0.93 (0.93, 0.94)	0.95 (0.94, 0.96)
MCC	0.32 (0.28, 0.37)	0.26 (0.22, 0.29)	0.32 (0.28, 0.36)	0.28 (0.25, 0.34)
AUC	0.80 (0.78, 0.83)	0.75 (0.73, 0.79)	0.80 (0.78, 0.83)	0.80 (0.78, 0.83)

In the subgroup excluding frontal horn hematoma, both TS and expert EI achieved an AUC of 0.80 (95% CI 0.78–0.83). Expert measurements yielded an F1 score of 0.40 (95% CI 0.36–0.44), sensitivity of 0.50 (95% CI 0.45–0.55), and specificity of 0.88 (95% CI 0.87–0.89), whereas TS measurements yielded an F1 score of 0.36 (95% CI 0.33–0.39), sensitivity of 0.70 (95% CI 0.63–0.74), and specificity of 0.73 (95% CI 0.71–0.75). Because the proportion of patients undergoing EVD was low, F1 scores and Matthews correlation coefficients were modest overall; detailed results are provided in Table 4 and Figure 7.

To further account for the multifactorial nature of EVD decision-making, multivariable logistic regression analyses were performed with adjustment for age, sex, admission GCS score, Hunt–Hess grade, and modified Fisher grade. Both TS- and expert-derived EI remained independently associated with EVD placement after covariate adjustment. The adjusted OR for TS-derived EI was 1.09 (95% CI 1.03–1.17, *p* = 0.009), whereas the adjusted OR for expert-derived EI was 1.23 (95% CI 1.11–1.37, *p* < 0.001). Among the included clinical covariates, only modified Fisher grade remained independently associated with EVD placement (Table 5).

**Table 5:** Multivariable logistic regression analysis for prediction of EVD placement using automated and expert Evans index measurements.

Variable	TS		Expert	
Term	OR	*p* value	OR	*p* value
Age	1.01 (0.97–1.05)	0.664	1.00 (0.97–1.04)	0.919
Gender	0.96 (0.46–1.98)	0.920	0.74 (0.34–1.57)	0.438
GCS	0.91 (0.76–1.08)	0.265	0.93 (0.78–1.12)	0.456
Hunt-Hess	1.52 (0.80–2.93)	0.204	1.54 (0.80–3.01)	0.195
Modified fisher	2.13 (1.33–3.74)	0.004 **	2.10 (1.29–3.71)	0.005 **
EI	1.09 (1.03–1.17)	0.009 **	1.23 (1.11–1.37)	<0.001 ***

### 3.6 Discrepancy analysis between TS and expert EI measurements

**Figure 8:**
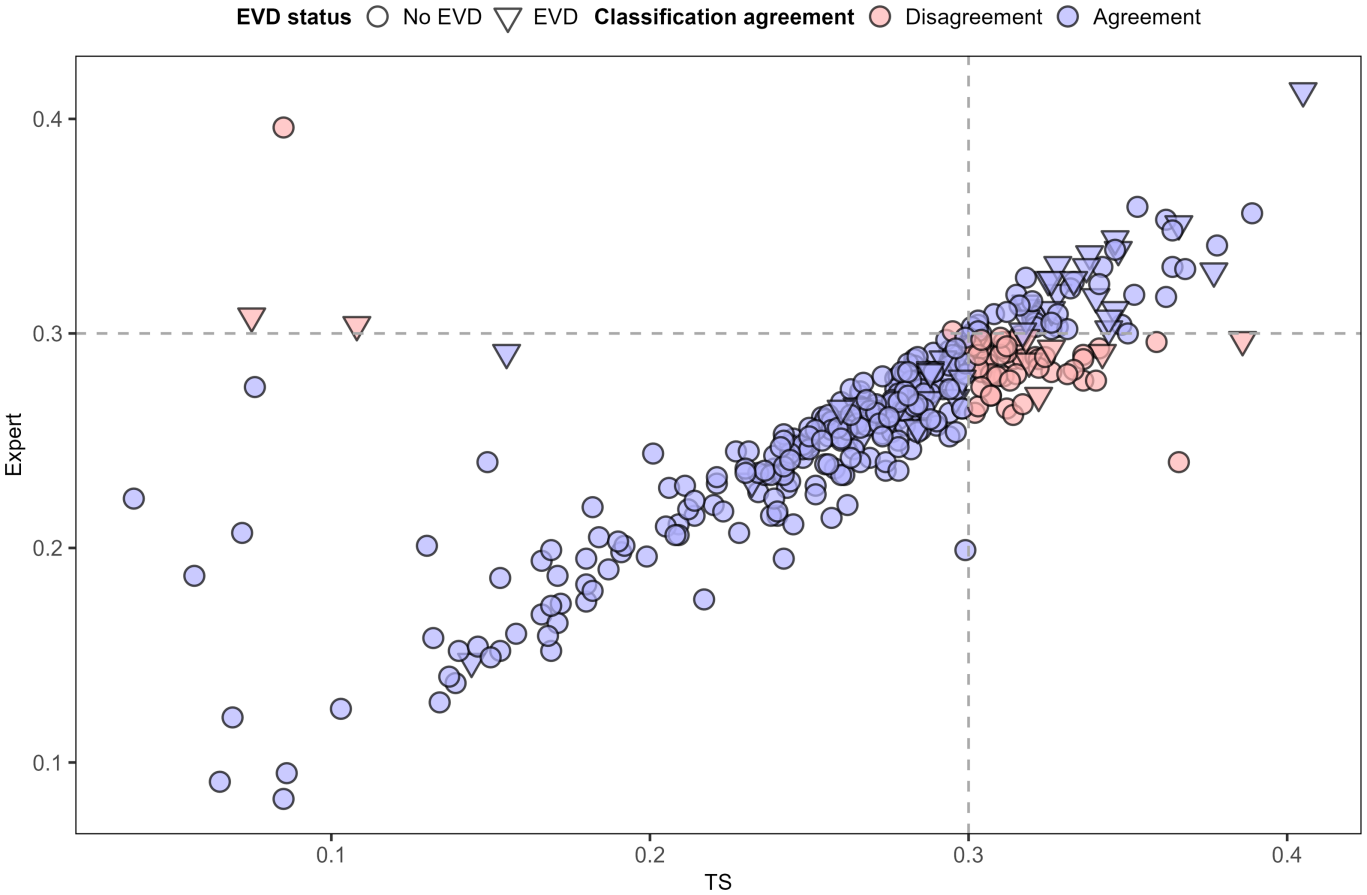
Agreement between automated and expert Evans index measurements stratified by EVD status. Scatter plot shows the relationship between Evans index (EI) values measured by TotalSegmentator (TS) and expert assessment. Each point represents one patient. Point shape indicates external ventricular drainage (EVD) status (circles, No EVD; triangles, EVD), and point color denotes classification agreement or disagreement between TS and expert measurements based on the predefined EI threshold of 0.30.

There was a certain degree of discrepancy between TS- and expert-derived EI measurements (Figure 8). Retrospective case-level review of scans with substantial disagreement between TS and expert measurements (absolute EI difference > 0.05) demonstrated distinct directional discrepancy patterns across imaging features (Table 6). Frontal horn hemorrhage was predominantly associated with TS underestimation of EI (15 cases: 14 underestimations and 1 overestimation), whereas frontal horn periventricular edema adjacent to the frontal horns was primarily associated with TS overestimation (22 cases: 20 overestimations and 2 underestimations).

**Table 6:** Imaging features identified by expert case-level review in cases with large discrepancies between TS and expert Evans index measurements.

Feature	*N*	Overestimate (TS>Exp)	Underestimate (TS<Exp)
Frontal horn hemorrhage	15	1	14
Hemorrhage in other ventricular regions	5	2	3
Frontal horn periventricular edema	22	20	2
Intracerebral hemorrhage	8	3	5
Interhemispheric fissure hematoma	7	2	5

Additional imaging findings, including intracerebral hemorrhage, interhemispheric fissure hematoma, and hemorrhage in other ventricular regions, were identified less frequently and demonstrated more heterogeneous discrepancy patterns without a consistent directional tendency.

## 4 Discussion

In this study, we systematically evaluated TotalSegmentator (TS), an open-source deep learning pipeline, for automated Evans index (EI) measurement on admission CT in patients with spontaneous subarachnoid hemorrhage (SAH) (26–28). We focused on measurement reproducibility, agreement with expert assessment, and clinical relevance using external ventricular drainage (EVD) as a pragmatic intervention endpoint reflecting contemporaneous clinical decision-making. Overall, TS demonstrated excellent within-method reproducibility comparable to expert measurements, achieved good agreement with experts for continuous EI measurements in the overall cohort, and showed substantially improved agreement after exclusion of frontal horn hematoma or frontal horn periventricular edema. Automated EI measurements also captured clinically relevant imaging signals associated with immediate EVD placement, with discrimination approaching expert assessment and becoming comparable in the subgroup without frontal horn hematoma.

To our knowledge, this study represents one of the first systematic validations of an off-the-shelf, general-purpose, open-source deep learning model for EI measurement in the complex imaging context of acute SAH. Prior automated ventricular assessment studies have largely focused on more anatomically structured conditions, such as normal pressure hydrocephalus, chronic ventricular enlargement, or pediatric hydrocephalus (5, 29, 30), with some reporting moderately good diagnostic performance in selected populations (31–33). However, these studies primarily used ventricular volume or related metrics as endpoints, involved relatively limited sample sizes, rarely evaluated agreement under the complex hemorrhagic morphology encountered in acute SAH, and generally lacked validation against clinically relevant intervention decisions made in acute neurocritical care settings (32, 34, 35). By applying a standardized agreement framework in a moderate-sized SAH cohort and directly relating automated EI measurements to clinically performed EVD placement based on admission imaging, our study addresses an important gap regarding the clinical reliability and practical relevance of existing open-source models in emergency neuroimaging workflows.

High reproducibility is a prerequisite for any quantitative imaging tool intended for clinical application. TS produced highly stable EI values across repeated runs, with minimal measurement variability and reproducibility comparable to repeated expert measurements. This finding is clinically important because an automated tool lacking internal consistency cannot serve as a reliable standardized reference, even if it demonstrates acceptable correlation with human readers. Between-method agreement between automated and expert EI measurements in the overall cohort fell within the range typically considered “good” but did not reach thresholds commonly labeled as “excellent” (36). Importantly, expert measurement itself is not a perfect gold standard and remains susceptible to slice selection, hemorrhagic artifacts, and reader-dependent interpretation (37). Agreement metrics should therefore be interpreted within the context of SAH-specific imaging complexity rather than judged solely against benchmarks derived from anatomically regular conditions.

A key finding was the divergence between good continuous agreement and only moderate threshold-based agreement at the conventional EI > 0.30 cutoff. This apparent discrepancy was largely explained by threshold sensitivity. Disagreement-density analysis showed that discordant classifications were concentrated around the EI = 0.30 threshold, indicating that relatively small differences between automated and expert measurements were sufficient to alter binary classification regarding whether ventricular enlargement met a clinically actionable threshold at the time of imaging. Threshold sensitivity analysis further demonstrated that agreement metrics varied non-linearly across cutoffs and peaked at approximately EI = 0.32. However, although Cohen’s kappa, Gwet’s AC1, PABAK peaked around EI = 0.32, the conventional EI cutoff of 0.30 remains clinically interpretable and widely used; therefore, alternative thresholds should be considered exploratory rather than definitive. Increasing the threshold improved specificity but reduced sensitivity and positive classification prevalence, potentially reducing sensitivity for identifying patients with clinically significant ventricular enlargement at presentation.

The moderate Cohen’s kappa value should also be interpreted in the context of class imbalance. Positive EI classifications were relatively infrequent in this cohort, and Cohen’s kappa is known to be sensitive to prevalence imbalance. In contrast, Gwet’s AC1 and PABAK consistently demonstrated substantially higher agreement across analyses, supporting the interpretation that agreement was better than Cohen’s kappa alone might suggest. Therefore, the reduced Cohen’s kappa at EI > 0.30 likely reflects a combination of borderline threshold effects and prevalence-related statistical behavior rather than broad systematic failure of automated EI measurement.

The prespecified subgroup and discrepancy analyses clarified disease-specific sources of variability in automated EI assessment. Disagreement between TS and expert measurements was not random but followed identifiable imaging patterns. Frontal horn hemorrhage was predominantly associated with TS underestimation of EI, likely because hyperdense clot adjacent to the ventricular margin obscured the frontal horn boundary on CT imaging. In contrast, frontal horn periventricular edema was mainly associated with TS overestimation, probably reflecting partial inclusion of low-density periventricular tissue within the automated ventricular segmentation boundary. Other imaging findings, including intracerebral hemorrhage, interhemispheric fissure hematoma, and hemorrhage in other ventricular regions, were less frequent and demonstrated heterogeneous directional patterns without consistent over- or underestimation tendencies.

The subgroup analyses were consistent with these failure-mode observations. Excluding frontal horn hematoma substantially improved continuous agreement between TS and expert measurements, suggesting that clot-related obscuration of ventricular boundaries is an important source of measurement variability. By contrast, excluding frontal horn periventricular edema had relatively little effect on continuous agreement but improved threshold-based classification metrics, indicating that edema-related segmentation effects were most relevant in borderline cases near the EI = 0.30 cutoff. Specifically, Cohen’s kappa, Gwet’s AC1, and PABAK improved after exclusion of periventricular edema, whereas ICC and Lin’s CCC remained largely stable, suggesting greater sensitivity of threshold-based metrics to subtle boundary-related effects.

Beyond measurement agreement, we evaluated whether automated EI measurements captured clinically relevant ventricular status associated with real-world EVD decision-making at presentation. Both automated and expert EI demonstrated moderate discriminative ability in the overall cohort, with similar overall performance and nearly identical discrimination after exclusion of frontal horn hematoma. TS measurements showed a tendency toward higher sensitivity with somewhat lower specificity, consistent with a conservative screening profile favoring reduction of missed cases with clinically significant ventricular enlargement requiring urgent intervention. These findings suggest that, when considering imaging-derived EI alone, automated measurements by TS demonstrated performance broadly comparable to expert assessment for identifying patients likely to undergo clinically indicated EVD placement.

Nevertheless, EVD placement decisions in patients with SAH are inherently multifactorial and influenced by neurological status, hemorrhage burden, intracranial pressure, hydrocephalus severity, and overall clinical judgment at the time of evaluation. Accordingly, we performed adjusted multivariable analyses incorporating established clinical severity markers. Both TS-derived and expert-derived EI remained independently associated with EVD placement after adjustment for age, sex, admission GCS score, Hunt–Hess grade, and modified Fisher grade. Although expert-derived EI demonstrated a somewhat stronger adjusted association with EVD placement, TS-derived EI remained independently associated with clinically performed EVD placement, supporting the clinical relevance of automated EI assessment as an adjunctive marker of ventricular status during acute decision-making rather than as a standalone predictor of future intervention.

An important observation is that disagreement between automated and expert measurements did not necessarily reduce clinical discrimination for EVD placement. Agreement metrics quantify concordance around a fixed threshold, whereas EVD-related discrimination reflects the ability of imaging measurements to capture clinically meaningful ventricular changes present at the time of CT acquisition. Frontal horn periventricular edema—including interstitial edema and cerebrospinal fluid redistribution—is itself clinically relevant in acute hydrocephalus, as it may reflect trans ependymal cerebrospinal fluid flow and elevated intraventricular pressure. In our imaging review, such regions were a frequent source of automated EI overestimation due to partial inclusion of non-ventricular low-density tissue. However, because these imaging features are also associated with clinically significant hydrocephalus and urgent EVD requirement, their inclusion may paradoxically preserve the ability of automated EI to identify clinically severe cases despite disagreement relative to expert measurements. This mechanism likely contributes to the observed pattern of higher sensitivity but lower specificity for automated EI in EVD discrimination analyses.

EI, whether automated or manual, should not be used as the sole criterion for EVD placement. Instead, automated EI may serve as a rapid and standardized adjunctive imaging biomarker for assessing ventricular enlargement at the time of presentation, particularly in emergency, resource-limited, or non-specialized settings where rapid objective quantification may support triage and immediate neurocritical care evaluation. Acute hydrocephalus after SAH is multifactorial, involving ventricular morphology, hemorrhage burden, edema, and intracranial pressure dynamics. The present study validates an open-source automated EI pipeline and provides a foundation for future multimodal approaches integrating imaging and clinical parameters to improve real-time assessment of hydrocephalus severity and EVD requirement (38, 39).

Several limitations should be acknowledged. First, this was a single-center retrospective study with limited scanner diversity, which may constrain generalizability and warrants external multicenter validation. Second, TS was evaluated as an off-the-shelf model and was not fine-tuned for SAH-specific hemorrhagic morphologies; targeted retraining may improve robustness in cases with distorted frontal horn anatomy. Third, EVD placement was used as a pragmatic clinical endpoint reflecting real-world intervention decisions rather than a perfect objective reference standard for hydrocephalus severity. Finally, although the present study supports the measurement reliability and clinical relevance of automated EI assessment during acute clinical evaluation, prospective studies are needed to determine whether implementation of automated EI can improve workflow efficiency, triage performance, or early neurocritical care decision-making in routine clinical practice.

## 5 Conclusion

This study shows that automated Evans index measurement using an open-source deep learning pipeline is highly reproducible in patients with acute spontaneous subarachnoid hemorrhage and achieves good agreement with expert assessment. Although agreement is affected by hemorrhage-related anatomical distortion, particularly frontal horn hematoma, automated measurements retain clinically informative discrimination for external ventricular drainage. These findings support the use of open-source deep learning tools as standardized adjuncts for ventricular assessment in emergency neuroimaging and provide a foundation for further multicenter validation and model refinement.

## Data Availability

The raw data supporting the conclusions of this article will be made available by the authors, without undue reservation.
