# Supplementary material for: Automated Evans index measurement using deep learning in acute subarachnoid hemorrhage: reliability, agreement with experts, and association with external ventricular drainage
Source: Front Neurol. 2026 Jun 19;17:1786460. doi: 10.3389/fneur.2026.1786460 (PMC13330109; doi:10.3389/fneur.2026.1786460)
Supplement: Supplementary file 1 [file Data_Sheet_1.pdf]

Supplementary Material

1 Supplementary Materials and Methods

1.1 DICOM-to-NIfTI Conversion Procedure:

Original CT images were stored in Digital Imaging and Communications in Medicine (DICOM) format and converted to Neuroimaging Informatics Technology Initiative (NIfTI) format prior to automated image analysis. Conversion was performed using Mango software (version 4.1, build 1531).

For each patient, all available non-contrast head CT series were reviewed in Mango, and the thinnest available reconstructed CT series was selected for subsequent analysis according to the clinically available acquisition parameters. Depending on the original clinical imaging protocol, the selected slice thickness varied across patients and included 1 mm or 5 mm reconstructions. Selecting the thinnest available CT series for each patient helped preserve anatomical detail and improve the accuracy of ventricular segmentation and Evans index measurement.

After series selection, the CT images were exported directly from Mango in NIfTI (.nii) format using the software's built-in export function. The converted NIfTI images were subsequently used as input for TotalSegmentator-based automated image processing and Evans index measurement.

2 Supplementary Results

2.1 Additional dataset details

All patients underwent non-contrast head CT scans using a GE 256-slice Revolution CT scanner, a Philips Ingenuity 64-slice (128-layer) spiral CT scanner, or a Toshiba 640-slice spiral CT scanner. The scanning range extended from the skull base to the vertex. Scanning parameters were as follows: tube voltage, 120 kV; tube current, 250–500 mA; slice thickness, 0.625–5 mm; interslice interval, 1–2 mm; pitch, 1.2; and matrix size, 512 × 512."

2.2 Inference time and hardware

Automated Evans index measurements were performed using TotalSegmentator (version 2.11.0) within a Conda-managed Python environment under Ubuntu 22.04 running in Windows Subsystem for Linux 2 (WSL2). The workstation was equipped with an Intel Core i5-12600KF CPU, 64 GB RAM, and an NVIDIA GeForce RTX 3060 GPU with 12 GB VRAM. GPU acceleration was enabled using CUDA 12.9 and PyTorch 2.6.0.

The average automated inference time was approximately 60 seconds per case, including image loading, segmentation inference, and Evans index calculation.
